# Supplementary material for: The Human Myofibroblast Marker Xylosyltransferase-I: A New Indicator for Macrophage Polarization
Source: Biomedicines. 2022 Nov 9;10(11):2869. doi: 10.3390/biomedicines10112869 (PMC9687871; doi:10.3390/biomedicines10112869)
Supplement: Supplementary file 1 [file biomedicines-10-02869-s001.zip › biomedicines-1970946-supplementary.pdf]

Supplementary Material

Table S1: Oligonucleotide sequences of the primer systems used for qRT-PCR analysis. T_A: Annealing temperature.

Gene	Oligonucleotide sequence (5′-3′)	T_A
ACTA2	GACCGAATGCAGAAGGAG<br>CGGTGGACAATGGAAGG	59 °C
B2M	TGTGCTCGCGCTACTCTCTCTT<br>CGGATGGATGAAACCCAGACA	59 °C
CD206	TGCTACTGAACCCCCACAAC<br>ACCAGAGAGGAACCCATTCG	63 °C
HSPG2	TGAACCCACAGCGAAAC<br>GTGTAGGAGAGGGTGTATC	59 °C
IL1B	ACAGATGAAGTGCTCCTTCCA<br>GTCGGAGATTCGTAGCTGGAT	63 °C
IL8	GAACTGAGAGTGATTGAGAGTGGA<br>CTCTTCAAAAACTTCTCCACAACC	63 °C
RPL13A	CGGAAGGTGGTGGTCGTA<br>CTCGGGAAGGGTTGGTGT	63 °C
SDHA	AACTCGCTCTTGGACCTG<br>GAGTCGCAGTTCCGATGT	63 °C
SDC2	GGAGCTGATGAGGATGTA<br>AATGACAGCTGCTAGGAC	59 °C
SMAD7	AGATGCTGTGCCTTCCTC<br>GTCTTCTCCTCCCAGTATGC	63 °C
TGFB1	GCGATACCTCAGCAACC<br>ACGCAGCAGTTCTTCTCC	59 °C
XYLT1	GAAGCCGTGGTGAATCAG<br>CGGTCAGCAAGGAAGTAG	63 °C
XYLT2	ACACAGATGACCCGCTTGTGG<br>TTGGTGACCCGCAGGTTGTTG	63 °C

A

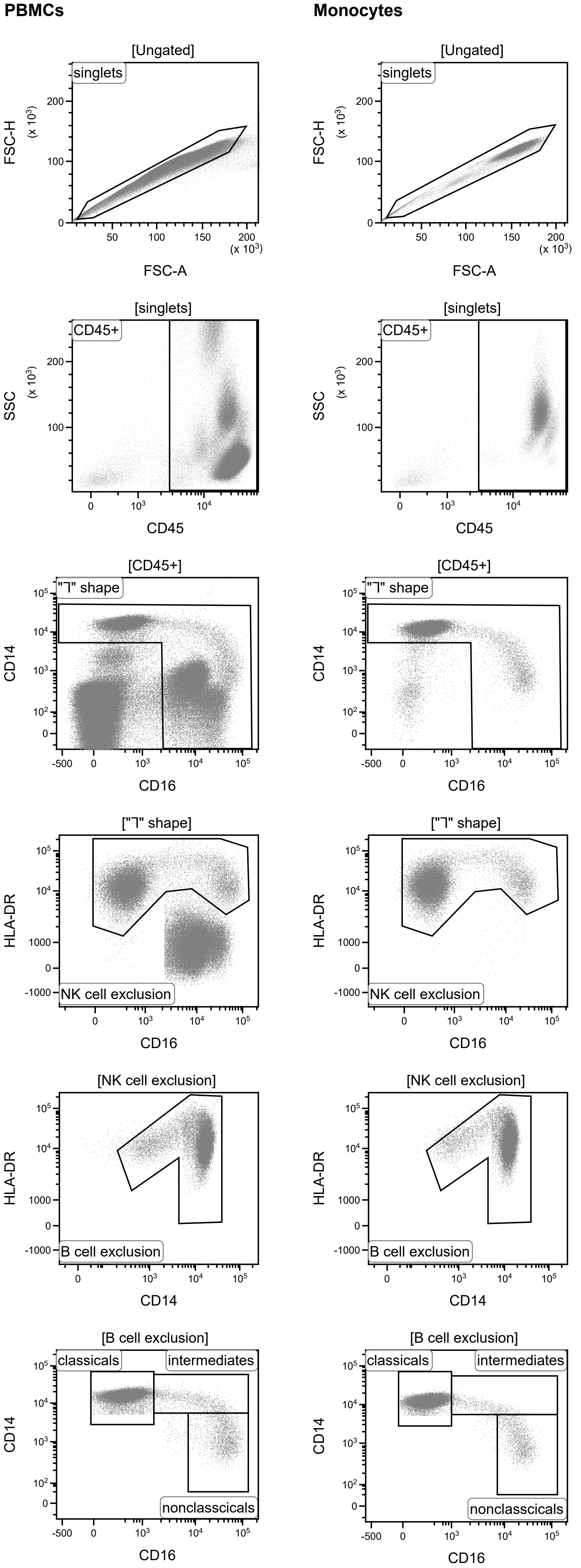

B

PBMCs

Gate	%Gated
All	100.00
singlets	94.90
CD45+	98.23
"⅂" shape	26.81
NK cell exclusion	34.36
B cell exclusion	99.33
classicals	85.32
intermediates	5.45
nonclasscicals	8.73

Monocytes

Gate	%Gated
All	100.00
singlets	86.49
CD45+	93.74
"⅂" shape	92.84
NK cell exclusion	99.22
B cell exclusion	99.22
classicals	90.20
intermediates	2.93
nonclasscicals	6.52

Figure S1. Gating strategy for the characterization of monocytes after PBMC isolation from buffy coats or negative selection. The strategy was adopted with minor modifications from Marimuth et al. [7]. (**A**) $CD14^+$ and $CD16^+$ monocytes were isolated from $CD45^+$ singlets in the characteristic "Ꞁ" shape. The NK cells (HLA-DR- in HLA-DR/CD16 plot) and B cells ($HLA\text{-}DR^{high}/CD14^{low}$) were excluded. Finally, all types of monocytes, classical ($CD14^{++}/CD16^{-}$), intermediate ($CD14^{++}/CD16^{+}$) and nonclassical ($CD14^{low}/CD16^{++}$), were found in both samples. (**B**) Quantification of gated macrophage subsets.

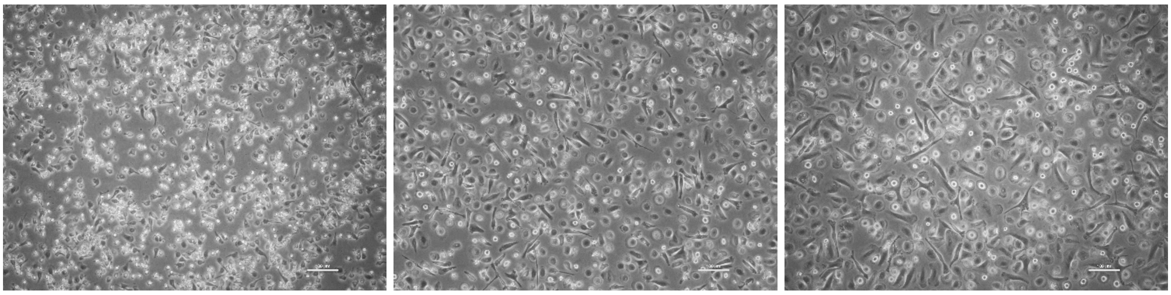

Figure S2. Phenotype of monocyte cultures matured to M0 M-MΦ. Representative images of negatively selected monocyte cultures on differentiation day 3, 6 and 8 (from left to right) using M-CSF for the *in vitro* generation of M-MΦ.

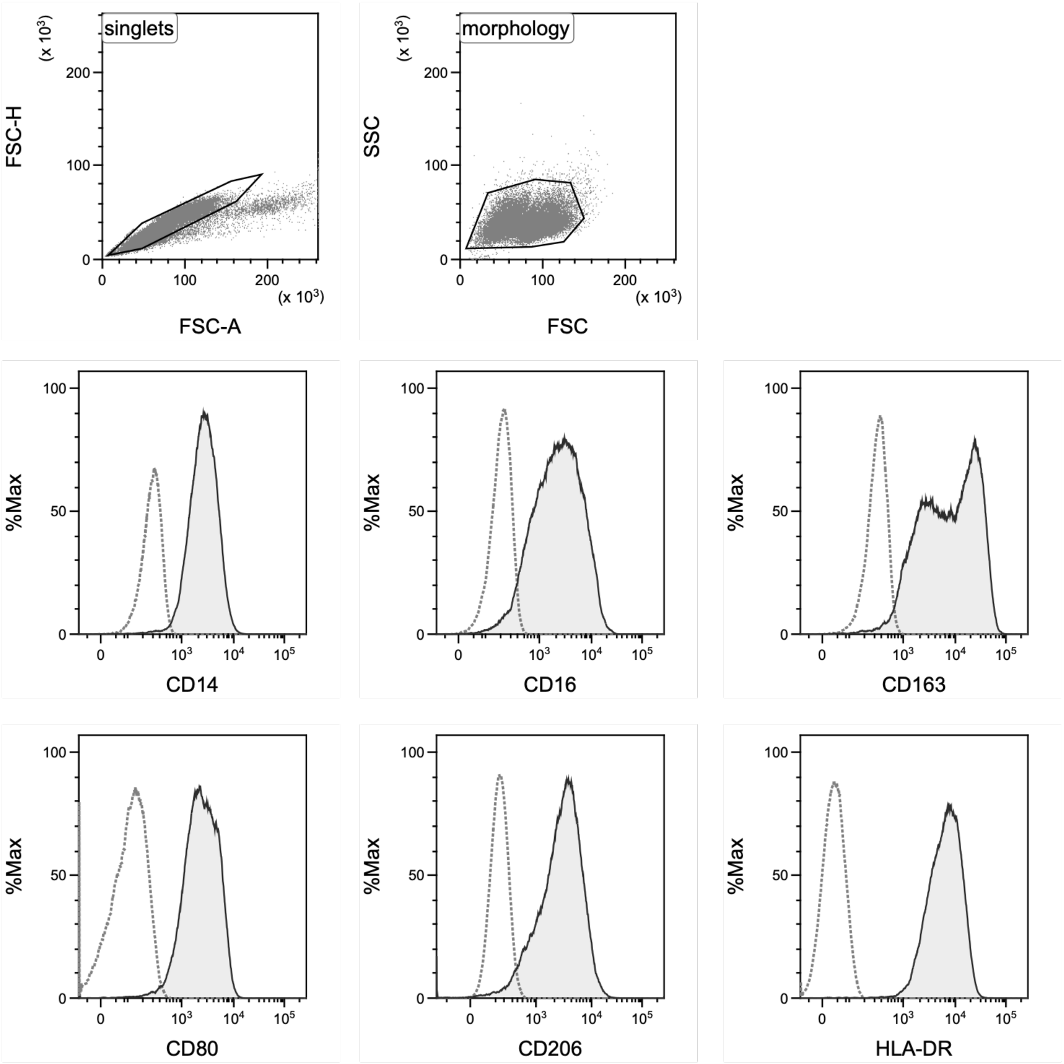

Figure S3. Gating strategy for the quantification of surface markers illustrated by the example of M0 M-MΦ. After doublet discrimination, single cells were gated based on morphology using forward scatter (FSC) and side scatter (SSC). The arithmetic mean fluorescence intensity (MFI) was measured (solid line) for the markers (CD14, CD16, CD80, CD163, CD206, and HLA-DR) and the MFI of unstained cells (dashed line) was subtracted (ΔMFI).

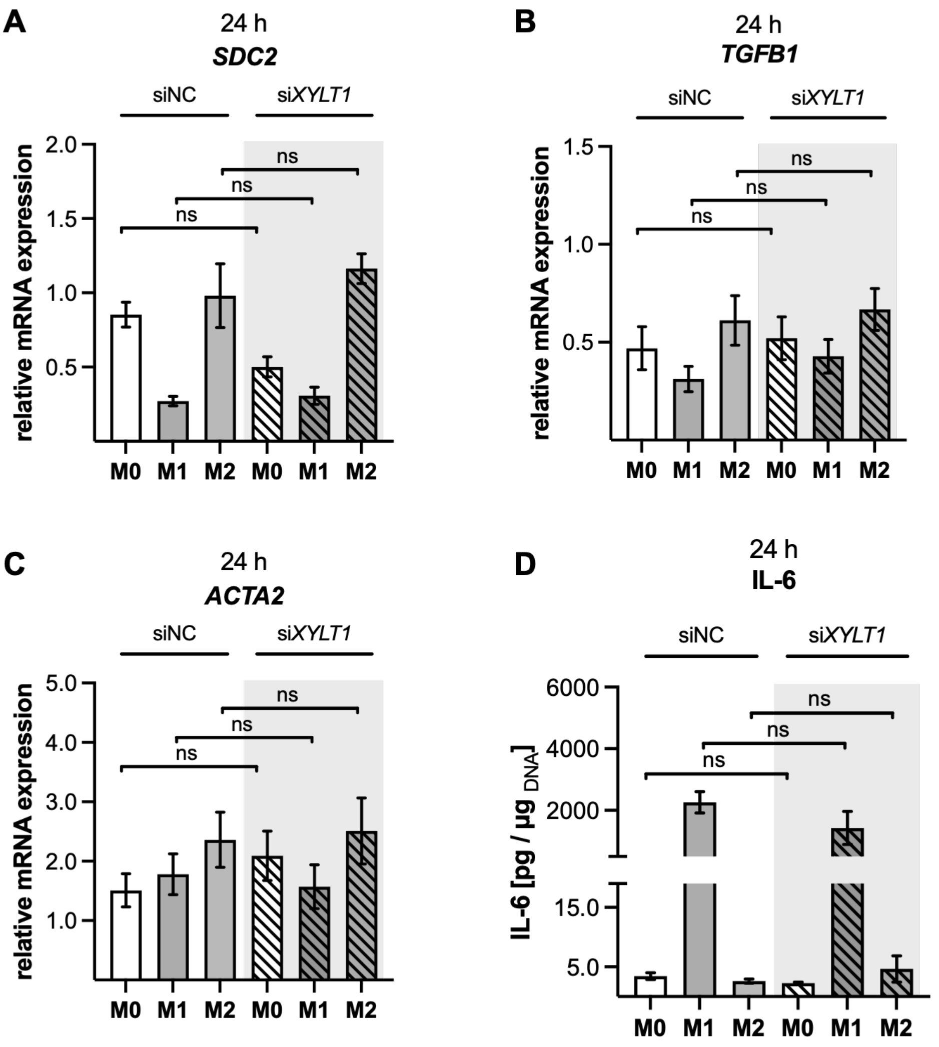

Figure S4. The mRNA expression of *SDC2*, *TGFB1* and *ACTA2* and protein level of IL-6 after siRNA-mediated *XYLT1* knockdown in polarized M-MΦ. Monocytes were differentiated to macrophages using M-CSF. On day 5, MΦ were treated with a non-targeting negative control siRNA (siNC) or a siRNA targeting *XYLT1* (si*XYLT1*). On day 6, MΦ were stimulated with IFN-γ/LPS (M1), IL-4 (M2) or no additive (M0). Cells were harvested after a polarization time of 24 h to determine the relative gene expressions of (**A**) *SDC2*, (**B**) *TGFB1* and (**C**) *ACTA2* by qRT-PCR and quantify the secretion of (**D**) IL-6 by immunoassay. The data (n, sample = 3; n, biological = 2; n, technical = 3) were analyzed using a one-way ANOVA, followed by Tukey's multiple comparison tests, and expressed as mean ± SEM. ns (not significant).
